# 3D Printed Personalized External Aortic Root Model in Marfan Syndrome with Isolated Sinus of Valsalva Aneurysm Caused by a Novel Pathogenic *FBN1* p.Gly1127Cys Variant

**DOI:** 10.3390/diagnostics11061057

**Published:** 2021-06-08

**Authors:** Jung Sun Cho, Joonhong Park, Jong Bum Kwon, Dae-Won Kim, Mahn-Won Park

**Affiliations:** 1 Department of Cardiology, College of Medicine, The Catholic University of Korea, Seoul 06591, Korea; tworugi@daum.net (J.S.C.); mirinesilver@catholic.ac.kr (D.-W.K.); 2 Department of Laboratory Medicine, Jeonbuk National University Medical School and Hospital, Jeonju 54907, Korea; miziro@jbnu.ac.kr; 3 Research Institute of Clinical Medicine of Jeonbuk National University-Biomedical Research Institute of Jeonbuk National University Hospital, Jeonju 54907, Korea; 4 Department of Thoracic and Cardiovascular Surgery, College of Medicine, The Catholic University of Korea, Seoul 06591, Korea; jbkwon@catholic.ac.kr

**Keywords:** 3D printed personalized model, aortic root replacement, Marfan syndrome, isolated sinus of Valsalva aneurysm, *FBN1* p.Gly1127Cys variant, Sanger sequencing

## Abstract

The major cause of death in Marfan syndrome (MFS) is cardiovascular complications, particularly progressive dilatation of the proximal aorta, rendering these patients at risk of aortic dissection or fatal rupture. We report a 3D printed personalized external aortic root model for MFS with an isolated sinus of Valsalva aneurysm caused by a novel pathogenic *FBN1* variant. A 67-year-old female with a history of lens dislocation and retinal detachment in the left eye was admitted for the evaluation of resting dyspnea several months prior. Transesophageal and transthoracic echocardiography revealed severe aortic valve regurgitation and a large left coronary sinus of Valsalva aneurysm in the proband. Sanger sequencing identified a heterozygous p.Gly1127Cys variant in the *FBN1* gene; previously, a mutation at this amino acid position was described as pathogenic (p.Gly1127Ser; rs137854468). A 3D printed personalized external aortic root model based on a multidetector computed tomography scan was constructed to illustrate the location of the ostium of the left main coronary artery on the aneurysm of the left coronary artery cusp. Aortic root replacement with the Bentall procedure matched the exact shape of the 3D printed model. Creation of a 3D printed patient-specific model could be useful in facilitating the development of next-generation medical devices and resolving the risks of postoperative complications and aortic root disease.

## 1. Introduction

Marfan syndrome (MFS, OMIM #154700) is an autosomal dominant systemic disorder that affects connective tissue, primarily in the skeletal (long limbs and fingers, scoliosis, and pectus deformities), ocular (ectopia lentis), and cardiovascular (aortic aneurysm and dissection) systems. MFS is caused by the fibrillin1 gene (*FBN1*), which is the primary disease-associated gene of MFS [1]. In approximately three-quarters of individuals with MFS, there is an affected parent. In a quarter of individuals, MFS is due to de novo pathogenic *FBN1* variants, with no differences according to sex, ethnicity, or geographical location [2]. Clinical manifestations of MFS are highly variable with a broad range of overlapping phenotypes, even among members of the same family [3]. Up to 97% of MFS patients who fulfilled the revised Ghent criteria for clinical diagnosis have *FBN1* mutations encoding a major component of the extracellular matrix microfibril, namely, fibrillin-1 [4]. There are at least 3000 described pathogenic variants of *FBN1*, and the disease phenotype depends on the type of mutation involved and its effect on the fibrillin-1 protein [5]. Dominant-negative (DN) missense mutations can lead to abnormal fibrillin-1 function that results from structurally altered fibrillin, which is incorporated together with normal fibrillin-1 derived from the nonmutated allele [3]. Mortality and morbidity are associated, above all, with aortic involvement including dilation, dissection, or rupture [6]. Skeletal, aortic, and pulmonary complications are more prevalent in adults with MFS than in children. Additionally, adult males are more likely to develop aortic root dilatation than females, and they more often need prophylactic aortic replacement [7]. Particularly, among MFS with a typical aortic root aneurysm, standard surgical management of aortic root aneurysm in MFS is considered to be either total root replacement or valve-sparing root replacement [8].

In this report, we described a Korean patient in which a novel p.Gly1127Cys variant in *FBN1* cosegregates with isolated left coronary sinus of Valsalva aneurysm and additional manifestations of lens dislocation with minor skeletal involvement. Furthermore, we applied a three-dimensional (3D) printed personalized external aortic root model to understand abnormal aortic structure in a patient with MFS.

## 2. Case Presentation

**Figure 1:**
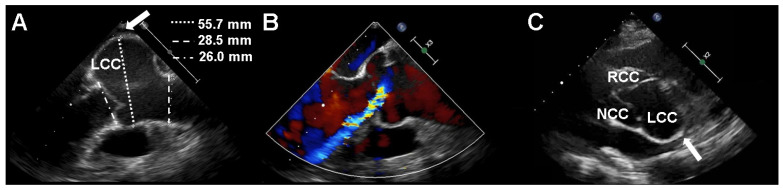
Transesophageal echocardiography (TEE) and transthoracic echocardiography (TTE) in the proband with Marfan syndrome. TEE (**A**) and (**B**) and TTE (**C**) revealed an isolated sinus of Valsalva aneurysm of the left coronary cusp (arrow) with severe aortic regurgitation. LCC, left coronary cusp; RCC, right coronary cusp; NCC, noncoronary cusp.

A 67-year-old female was admitted to the Department of Cardiology, Daejeon St. Mary’s Hospital (Daejeon, Korea) for the evaluation of resting dyspnea several months prior. She grew up in an orphanage when she was young and had no information about her parents. During childbirth, she developed a lens dislocation in her left eye. Lens removal surgery was performed but caused retinal detachment that led to blindness in the left eye after several ophthalmic surgeries. She had glaucoma in the right eye. She was 158 cm tall, but she had a slender build and arachnodactyly and a positive thumb sign. Transesophageal echocardiography (TEE) and transthoracic echocardiography (TTE) were performed to evaluate dyspnea and revealed severe aortic valve regurgitation (AR) and a large left coronary sinus of Valsalva aneurysm in the proband (Figure 1A–C). The left ventricular end-diastolic and left ventricular end-systolic dimensions were 57 and 41 mm, respectively. The left ventricular ejection fraction was 56%. Multi-detector computed tomography (MDCT) demonstrated that the maximal diameter of the sinus of Valsalva aneurysm was 64 mm and that the coronary artery was patent in the proband.

**Figure 2:**
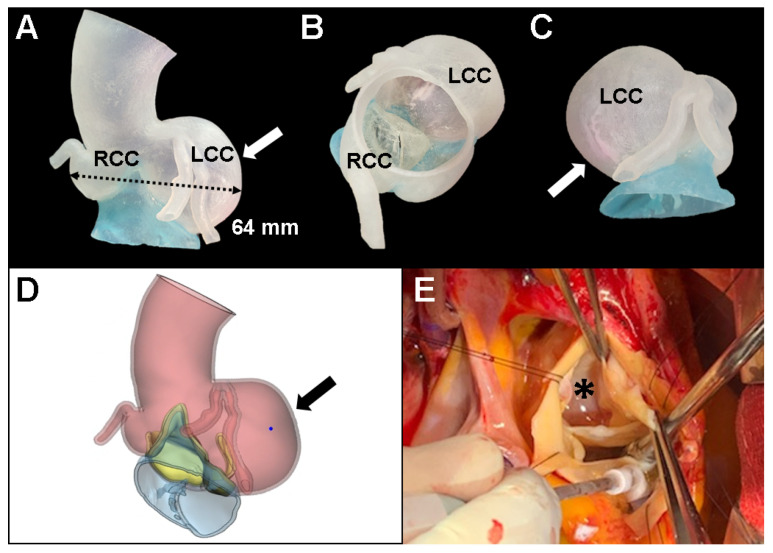
3D printed model in the proband with Marfan syndrome. To help understand the anatomic structure before surgery, a 3D printed personalized external aortic root model (**A**–**D**) was derived from multi-detector computed tomography to illustrate the location of the ostium of the left main aneurysm of the left coronary cusp (arrow). (**E**) Aortic root replacement with the Bentall procedure matched the exact shape of the 3D printed model (asterisk). LCC, left coronary cusp; RCC, right coronary cusp; NCC, noncoronary cusp.

The proband was going to undergo aortic root replacement (ARR) with the Bentall procedure due to the isolated left coronary sinus of Valsalva aneurysm and severe AR. To help understand the anatomic structure before the Bentall procedure, a 3D printed personalized external aortic root model based on the MDCT scan was constructed to illustrate the location of the ostium of the left main coronary artery on the aneurysm of the left coronary artery cusp. The patient’s CT datasets were imported into a medical 3D image-based engineering software (Materialize Mimics ver. 22 and 3-Matic ver. 13, Materialise NV, Leuven, Belgium) and the 3D model was printed on a Stratasys Connex3 Objet500 3D printer (Stratasys Ltd., Eden Prairie, MN, USA) using the form2 clear resin (FLGPCL02). At first, we considered valve sparing ARR for this case because aortic leaflets were relatively preserved even though annular dilatation was combined (26 mm). However, 3D printed model revealed that thinning of the cusp due to asymmetrically severely enlarged left coronary cusp can cause stress fenestration toward the commissures and turbulent blood flow inside the aorta (Figure 2A–D). Proband’s surgical anatomic model and 3D printed model were exactly matched (Figure 2E). Finally, she underwent a modified Bentall operation with a 21 mm mechanical aortic valve and left and right coronary artery buttons successfully.

**Figure 3:**
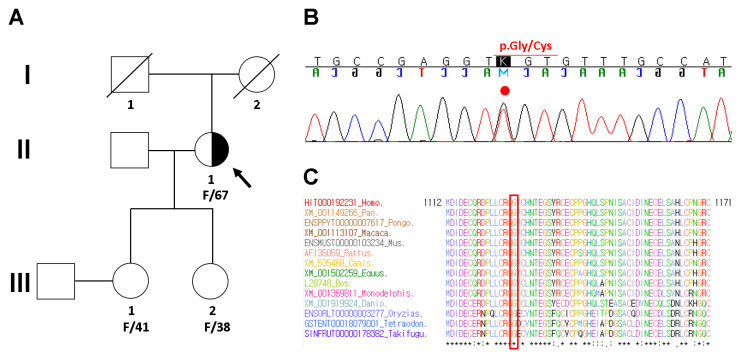
Pedigree analysis and sequencing results. (**A**) Pedigree of the proband (arrow) with Marfan syndrome caused by a heterozygous *FBN1* variant and her healthy family members. (**B**) Sanger sequencing revealed a heterozygous missense variant (NM_000138.4: c.3379G>T; p.Gly1127Cys) of the *FBN1* gene in the proband. The variant is highlighted by the red dot. (**C**) Sequence alignment of the conserved calcium binding epidermal growth factor (cbEGF)-like domain of the FBN1 protein in multiple vertebrate species. The protein sequence of the Gly1127 residue is highly conserved across the compared vertebrate species. It is highlighted in the red box.

To identify the potential genetic cause of MFS in the proband (II-1 in Figure 3A), Sanger sequencing was recommended. Genomic DNA was extracted from peripheral blood using a QIAamp DNA Mini Kit (Qiagen GmbH, Hilden, Germany). Entire coding exons and flanking intronic sequences of the *FBN1* gene were amplified by polymerase chain reaction using the primers described previously [9]. Sanger sequencing of PCR products was performed using the BigDye Terminator v3.1 Cycle Sequencing Kit (Applied Biosystems, Foster City, CA, USA) and was resolved by capillary electrophoresis on an 3730XL Genetic Analyzer (Applied Biosystems, Carlsbad, CA, USA). Electropherograms were interpreted using Sequencher DNA Sequence Analysis Software Ver 4.9 (Gene Codes Corporation, Ann Arbor, MI, USA). All identified variants were confirmed by bidirectional resequencing. The *FBN1* sequence with RefSeq ID NM_000138.4 was used as a reference for cDNA nucleotide numbering. Mutations predicted to cause strong and moderate alterations in gene functions were manually reviewed by laboratory geneticists based on ACMG-AMP standards and guidelines [10]. As a result, Sanger sequencing identified a heterozygous c.3379G>T mutation, resulting in an amino acid change of glycine to cysteine at position 1127 (NM_000138.4: c.3379G>T; p.Gly1127Cys) in the *FBN1* gene (Figure 3B). Another mutation at the same amino acid position had previously been described as pathogenic (NM_000138.4: c.3379G>A; p.Gly1127Ser; rs137854468) [11]. Sequence alignment of the conserved calcium binding epidermal growth factor (cbEGF)-like domain of the FBN1 protein demonstrated that the Gly1127 residue is highly conserved across multiple vertebrate species (Figure 3C).

In approximately 75% of individuals with MFS, the syndrome is inherited in an autosomal dominant manner from an affected parent [12]. Thus, genetic counseling and segregation analysis were recommended for the family members of the proband. She had two daughters aged 41 and 38, and no MFS clinical symptoms were found during their echocardiography or physical examination. They refused consent for the genetic test, and genetic counseling and segregation analysis were not evaluated.

## 3. Discussion

Mutated fibrillin-1 results in dysregulation of TGF-β signaling as well as structural weakness of connective tissue, both of which contribute to complicated pathogenesis in MFS [13]. It plays a communicative role in biosignaling by regulating the local bioavailability of TGF-β, and it plays a role in mechanosignaling by interacting with mechanosensors and providing feedback to regulate the response to hemodynamic changes [14]. *FBN1* mutations have been associated with a broad spectrum of phenotypes in classic MFS, and while some correlations between the *FBN1* genotype and clinical phenotype are known, few are predictive [15]. The correlation between *FBN1* genotypes and phenotypes has been extensively reported, and more than 3000 pathogenic mutations are distributed throughout the entire length of the gene [3,5,15,16]. *FBN1* variants with cysteine (Cys) substitutions are more frequently associated with a high prevalence of ectopia lentis such as partial or complete displacement of the eye lens [17]. *FBN1* mutations in exons 24–32 define a high-risk group for cardiovascular manifestations at all ages [15]. MFS with haploinsufficient type *FBN1* variants such as nonsense and out-of-frame variants that presumably cause nonsense-mediated mRNA decay have more severe aortic phenotypes than those with DN-type mutations such as missense and in-frame variants that are expected to exert loss-of-function effects [3,5,16,17]. Deleterious variants among DN patients showed that patients with mutations affecting or creating cysteine residues and in-frame deletion variants in the cbEGF domains of exons 25–36 and 43–49 (DN-CD variants) had a 6.3-fold higher risk for aortic events than DN-nonCD patients, which is comparable to or more deleterious than haploinsufficient variants [3]. In our case, a novel missense p.Gly1127Cys mutation was identified at the same position in the *FBN1* gene as others that have caused different substitutions of the Gly1127 residue. The proband had complete displacement of the left eye lens during childbirth, and subsequent retinal detachment and total blindness developed. She had glaucoma in the right eye. She suffered from all kinds of ocular complications of MFS. The pathogenic criterion for this novel variant is weighted as moderate 5 (PM5) based on ACMG-AMP standards and guidelines [10]. A novel missense amino acid change occurring at the same position as another pathogenic missense change is considered moderate evidence but cannot be assumed to be pathogenic. This is especially true if the novel change is more conservative than the established pathogenic missense variant. Additionally, the different amino acid changes could lead to a different phenotype [10]. Francke and colleagues [11] reported that a Gly1127Ser mutation in the cbEGF-like domain of the *FBN1* gene produces a mild form of autosomal dominantly inherited weakness of elastic tissue, which predisposes patients to ascending aortic aneurysm and dissection later in life. Additionally, in our case, the proband had an isolated left coronary sinus of Valsalva aneurysm without dissection in late life. Recently, Hernándiz A and colleagues [18] reported evidence of the different clinical behaviors of patients with either a DN effect or haploinsufficiency of the *FBN1* variants and of males versus females. In addition, the observation of a “Carter effect” in the cardiovascular phenotype provides further evidence of the existence of inherited modifiers and supports the high heritability of cardiovascular clinical features [19].

The major cause of death in MFS is cardiovascular complications, particularly progressive dilatation of the proximal aorta, rendering these patients at risk of aortic dissection or fatal rupture [20]. Although the aortic sinus is most commonly affected, aneurysms and dissections in more distal aortic regions and in extra-aortic arteries can also occur [21]. The progression of pulmonary artery dilatation is associated with the occurrence of type B dissection, which might reflect tissue vulnerability [22]. Current standard management is echocardiographic monitoring with a consideration to replace the ascending aorta, with or without aortic valve replacement. Prophylactic root replacement potentially neutralizes that hazard and is the recommended management for patients who meet the widely accepted criteria defining them as high risk [14]. Personalized external aortic root support is computer designed and manufactured to match the aortic root morphology of the individual patient based on multiple measurements of images before and after surgery. This approach is intended to prevent ongoing dilatation of an already morphologically abnormal aorta with the purpose of reducing the risk of death due to dissection in the aortic root [23]. The operation does not require opening the heart or aorta or any interference with cerebral or myocardial perfusion, and it is usually performed without the need for cardiopulmonary bypass. It is applicable to patients with root aneurysms caused by genetically determined etiologies such as MFS and Loeys-Dietz and Ehlers-Danlos syndrome [8]. Isolated left coronary sinuses of Valsalva aneurysms are extremely rare cardiovascular manifestations in patients with MFS, and few cases have been reported [24,25]. Aortic root 3D printing is an emerging technique and may improve the success rate and safety of aortic valve replacement [26,27]. Visualization via 3D printing can help surgeons and cardiologists understand the anatomy of rare aortic root disease and plan the procedure. In this case, we applied 3D printing, and the surgical findings of the proband matched the 3D printed model such as the location of the ostium of the left main coronary artery on the aneurysm of the left coronary artery cusp. Furthermore, patient-specific 3D printing models such as this case are expected to help surgeons reduce surgery time, side effects, and complications [26,27]. In our case, severe AR with aortic root aneurysm could be performed composite valve graft or valve sparing aortic root replacement. Valve sparing aortic root replacement can avoid anticoagulation and prosthesis-related complications. However, a composite valve graft was performed as a modified Bentall operation after analysis of the 3D model because of aortic annular dilatation (aortic annulus, 26 mm) and thinning of the cusps due to the asymmetrically severely enlarged left coronary cusp. Thinning of the cusps when involved with more than one cusp, valve sparing aortic root replacement is not advised. Additionally, more than 25 mm annular dilatation was a risk factor of early and late failure [28].

## 4. Conclusions

In conclusion, we reported a 3D printed personalized external aortic root model in MFS with an isolated left coronary sinus of Valsalva aneurysm caused by a novel pathogenic *FBN1* p.Gly1127Cys variant. Creation of a 3D printed patient-specific model could be useful in facilitating the development of next-generation medical devices and resolving the risks of postoperative complications and aortic root disease. Genetic analysis of the *FBN1* gene is essential to discover its genetic origin in MFS patients with isolated left coronary sinus sinuses of Valsalva aneurysm.

## Data Availability

The data presented in this study are available upon request from the corresponding author.
